# Clock gene *Bmal1* is dispensable for intrinsic properties of murine hematopoietic stem cells

**DOI:** 10.1186/1477-5751-13-4

**Published:** 2014-03-08

**Authors:** Aki Ieyasu, Yoko Tajima, Shigeki Shimba, Hiromitsu Nakauchi, Satoshi Yamazaki

**Affiliations:** 1 Laboratory of Stem Cell Therapy, Center for Experimental Medicine, the Institute of Medical Science, the University of Tokyo, Tokyo 108-8639, Japan; 2 Department of Health Science, School of Pharmacy, Nihon University, Funabashi, Chiba 274-8555, Japan; 3 Current address: 4-6-1 Shirokanedai, Minato-ku, Tokyo 108-8639, Japan

**Keywords:** Hematopoietic stem cells, Cell cycle, Circadian rhythm, Bmal1 and *Bmal1*^−/−^ mice

## Abstract

**Background:**

Circadian rhythms are known to influence a variety of biological phenomena such as cell cycle, sleep-wake rhythm, hormone release and other important physiological functions. Given that cell cycle entry of hibernating hematopoietic stem cells (HSCs) plays a critical role in controlling hematopoiesis, we asked functional significance of the clock gene *Bmal1*, which plays a central role in regulating circadian rhythms as a transcription factor. Here we investigated the necessity of Bmal1 for HSC functions using *Bmal1* deficient (*Bmal1*^−/−^) mice.

**Findings:**

Using colony-forming assays *in vitro,* we found that the frequency of mixed colony formation between *Bmal1*^*+/+*^ and *Bmal1*^*−/−*^ CD34^−^KSL cells does not differ significantly. Competitive bone marrow assays also revealed that *Bmal1*^−/−^ bone marrow cells competed normally with wild-type cells and displayed long-term multi-hematopoietic lineage reconstitution. In addition, there were no significant differences in the frequencies and hibernation state of bone marrow HSCs between *Bmal1*^*+/+*^ and *Bmal1*^−/−^ mice, suggesting that they are independent of circadian rhythms.

**Conclusions:**

This paper discusses the necessity of circadian rhythms for HSC functions. Our data clearly shows that a key circadian clock gene *Bmal1* is dispensable for intrinsic functions of HSCs, such as differentiation, proliferation and repopulating ability.

## Findings

### Background

Hematopoietic stem cells (HSCs) reside in specialized bone marrow (BM) microenvironments, called niches, providing the entire range of blood cells throughout the lifespan [1,2]. We have recently demonstrated that non-myelinating Schwann cells induce hibernation of HSCs in mouse BM [3,4]. Occasionally, most HSCs in the BM niche come out of hibernation and undergo cell division on average every one to two months [5,6]. Although the molecular mechanisms underlying re-entry into the cell cycle remain obscure, recent evidence suggests that the circadian clock regulates HSC trafficking between the BM and peripheral blood (PB) via the sympathetic nervous system [7]. To address the relationship between circadian oscillation and HSC hibernation in BM hematopoiesis, we here considered the possibility that the circadian transcription factor Bmal1 [8] is involved in BM hematopoiesis. Accumulating evidences have suggested that BMAL1 forms heterodimers with CLOCK, binds to E-box sequences in the promoter region and regulates the transcription of a number of clock-controlled genes. We therefore examined differentiation, proliferation and repopulating capacity of HSCs in Bmal1 deficient (*Bmal1*^*−/−*^) mice which demonstrate complete loss of circadian behavioral rhythms [9] and only half life span of wild-type mice [10]. Our findings led to the conclusion, however, that Bmal1 is dispensable for differentiation, proliferation and repopulating ability of murine HSCs.

## Materials and methods

### Mice

C57BL/6-Ly5.1 (B6-Ly5.1) and C57BL/6-Ly5.1/5.2-F1 (B6-F1) mice were purchased from Sankyo-Lab Service (Tsukuba, Japan). *Bmal1*^*−/−*^ mice were obtained by mating *Bmal1*^+/−^ mice [11] bred and maintained in the Animal Research Facility of the Institute of Medical Science, the University of Tokyo. Animal care in our laboratory was in accord with the guidelines of the University of Tokyo for animal and recombinant DNA experiments.

### CFU-Cs assay

PB mononuclear cells were isolated from 400 μl PB on Ficoll-Paque PLUS (GE Healthcare, Buckinghamshire, England) and CFU-Cs assays were performed using MethoCult (STEMCELL Technologies, Vancouver, Canada) according to manufacturer’s protocols. On day 11 of culture, colonies were observed under light microscopy.

### Purification of murine HSCs

Mouse CD34^−^KSL HSCs were purified from BM cells of 8-10-week-old mice. The cells were stained with an antibody cocktail consisting of biotinylated anti-Gr-1, −Mac-1, −CD4, −IL-7R, and -Ter-119 (eBioscience, San Diego, CA), and -B220 and -CD8 monoclonal antibodies (BioLegend, San Diego, CA) (lineage-marker cocktail). Lineage-positive cells were depleted with anti-Biotin MicroBeads (Miltenyi Biotec, Auburn, CA) and LS columns (Miltenyi Biotec). The remaining cells were further stained with fluorescein isothiocyanate (FITC)-conjugated anti-CD34 (BD Bioscience, California, CA), phycoerythrin (PE)-conjugated anti-Sca-1 (eBioscience), and allophycocyanin (APC)-conjugated anti-c-Kit antibodies (BioLegend). Biotinylated antibodies were detected with streptavidin-APC-Cy7 (BioLegend). Analysis and cell sorting were performed on a MoFlo using Summit software (Dako, Glostrup, Denmark) and results were analyzed with FlowJo software (Tree Star, Ashland, OR).

### Colony assays and single-cell cultures

CD34^−^KSL HSCs were clonally deposited into 96-well micro-titer plates containing 200 μl of S-Clone SF-03 (Sanko Junyaku Inc, Tokyo, Japan) supplemented with 10% BSA and cytokines (50 ng/ml mouse SCF, 50 ng/ml human TPO, 20 ng/ml mouse IL-3 and 2 U/ml mouse EPO for colony assays; 50 ng/ml mSCF, 50 ng/ml hTPO for proliferation assays). Colonies were recovered on day 11 of culture, cytospun onto slide glasses and subjected to Hemacolor staining (MERCK, Darmstadt, Germany) for morphological examination. To observe proliferation potential of CD34^−^KSL cells, cells were counted under light microscopy.

### Competitive repopulation assays

Competitive repopulation assays were performed using the Ly5 congenic mouse system. 1 × 10^6^ BM cells from *Bmal1*^+/+^ or *Bmal1*^−/−^ mice (B6-Ly5.2) and the same number of BM competitor cells from B6-F1 mice were transplanted into B6-Ly5.1 mice irradiated at a dose of 9.5 Gy. After transplantation, PB cells of the recipients were stained with PE-conjugated anti-Ly5.1 (BioLegend) and FITC-conjugated anti-Ly5.2 (BD Bioscience). The cells were further stained with PE-Cy7-conjugated anti-Mac-1 and -Gr-1, Pacific Blue (PB)-conjugated anti-B220 and APC-Cy7-conjugated anti-CD3 antibodies (BioLegend) and then analyzed on a FACS Aria (BD Bioscience). The second BMT was performed by transferring 1 × 10^6^ BM cells from femora and tibiae of the primary recipient mice into lethally irradiated Ly5.1 mice. PB cells from the secondary recipient mice were analyzed 4, 8 and 12 weeks after the second BMT.

### Cell cycle assays

To analyze the G_0_ phase, cells were incubated with 1 μg/ml Pyronin Y (Sigma-Aldrich, Saint Louis, Missouri) at 37°C for 30 min and analyzed on a FACS Aria. To investigate the turnover rate of CD34^−^KSL cells, EdU (invitrogen) was administered continuously to mice in the drinking water (0.5 mg/ml). After 3 weeks, BM cells were assessed with a Click-iT EdU PB Flow Cytometry Assay Kit (invitrogen) according to manufacturer’s protocols and analyzed on a FACS Aria.

### White blood cell differentiation

PB cells of 10 or 40-week-old *Bmal1*^+/+^ or *Bmal1*^−/−^ mice were stained with PE-conjugated anti-Gr-1, APC-conjugated anti-CD4, FITC-conjugated anti-CD8 (eBioscience), PE-Cy7-conjugated anti-Mac-1, PB-conjugated anti-B220 and APC-Cy7-conjugated anti-CD3 antibodies and then analyzed on a FACS Aria.

## Results and discussion

### *Bmal1*^*−/−*^ HSCs exhibit comparable differentiation and proliferation potentials *in vitro*

It has been shown that the mobilization of hematopoietic stem and progenitor cells (HSPCs) from BM is regulated by circadian clock [7]. We therefore considered the possibility that the circadian transcription factor Bmal1 is involved with BM hematopoiesis. Indeed we could detect oscillating CFU-Cs of HSPCs in PB of *Bmal1*^*+/+*^ mice at Zeitgeber time (ZT) 5 and ZT17, but there were no statistically significant fluctuations in case of *Bmal1*^*−/−*^ mice (Additional file 1: Figure S1A). Thus, oscillating CFU-Cs of HSPCs appear to be regulated by circadian clock, however, it is unclear how Bmal1 affects intrinsic functions of HSCs such as differentiation, proliferation and repopulating capacity. We therefore asked to investigate and clarify these problems.

**Figure 1:**
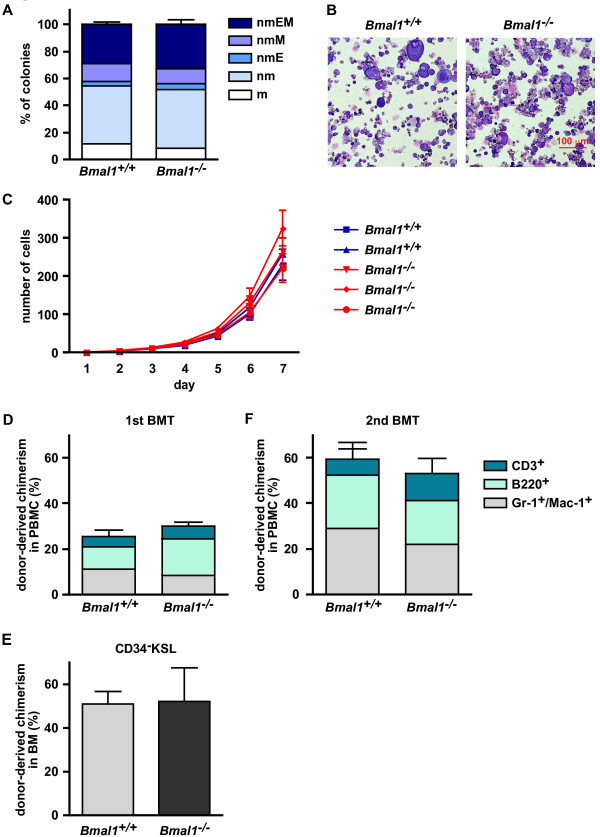
**Normal differentiation *in vitro *and normal long-term reconstitution ability *in vivo *of *Bmal1***^**−/− **^**HSCs. A, B)** Normal *in vitro* colony formation capacity of *Bmal1*^−/−^ HSCs. Single HSCs from *Bmal1*^+/+^ and *Bmal1*^−/−^ mice were cultured with cytokines for 11 days. Data shown are the mean numbers ± SDs of colonies of three independent experiments (n = 3). Colony cells were morphologically identified as neutrophils (n), macrophages (m), erythroblasts (E) and megakaryocytes (M). The scale bar in B is 100 μm. **C)** Comparable proliferation potentials of *Bmal1*^−/−^ HSCs. CD34^−^KSL HSCs were clonally deposited into 96-well micro-titer plates containing 200 μl of S-Clone SF-03 supplemented with 10% BSA and cultured with the indicated cytokines (50 ng/ml mouse SCF, 50 ng/ml TPO) for 7 days. Cell numbers were counted under a microscope. Data shown are mean numbers ± SEMs of colonies (n = 52). **D-F)** Comparable long-term reconstitution ability of *Bmal1*^+/+^ and *Bmal1*^−/−^ HSCs during serial transplantation. Lethally irradiated recipient B6-Ly5.1 mice were transplanted with 1 × 10^6^ BM cells (harvested at ZT5) from *Bmal1*^+/+^ and *Bmal1*^−/−^ mice (Ly5.2) and the same number of BM competitor cells from F1 mice in a competitive repopulation assay. Data shown are the mean ratios ± SDs of donor-derived cells in the PB at 12 weeks after the first BMT (**D**, n = 7), in the BM at 12 weeks after the first transplantation (**E**, n = 7), and in the PB at 12 weeks after the second BMT (**F**, n = 5) of three independent experiments.

For the present investigation of effects of *Bmal1* absence on differentiation of HSCs, we performed colony-forming assays *in vitro* in which freshly isolated *Bmal1*^*+/+*^ and *Bmal1*^*−/−*^ CD34^−^KSL cells were cultured for 11 days in medium supplemented with SCF, TPO, IL-3 and EPO. The resultant frequencies of mixed colonies (nmEM) did not differ significantly between *Bmal1*^*+/+*^ and *Bmal1*^*−/−*^ CD34^−^KSL cells (*Bmal1*^*+/+*^ CD34^−^KSL cells; 29.17 ± 1.18%, *Bmal1*^*−/−*^ CD34^−^KSL cells; 34.40 ± 2.22%) (Figure 1A). After close examination, we found that there is no significant morphological difference between the colonies of two groups (Figure 1B). In addition, *Bmal1*^*+/+*^ and *Bmal1*^*−/−*^ CD34^−^KSL cells demonstrated comparable proliferation potentials after 7 days culture (Figure 1C).

### Bmal1 is dispensable for Bone marrow reconstitution

To determine the repopulating ability of *Bmal1*^*−/−*^ HSCs *in vivo*, we designed a competitive repopulation assay. For this purpose, 1 × 10^6^ BM cells from *Bmal1*^*+/+*^ or *Bmal1*^*−/−*^ mice were transplanted into lethally irradiated recipient mice along with an equal number of BM cells from B6-F1 mice. At 4, 8 and 12 weeks after transplantation, flow cytometric analysis showed a high-level chimerism of B220^+^ cells in PB of the recipients transplanted with *Bmal1*^*−/−*^ BM cells, but this was not observed in the second Bone Marrow Transplantation (BMT). In addition, there was no statistically significant difference in the chimerism of Gr-1^+^/Mac-1^+^ and CD3^+^ cells. These results suggest that *Bmal1*^*+/+*^ and *Bmal1*^*−/−*^ BM cells are equally capable of hematopoietic reconstitution (Figure 1D and Additional file 1: Figure S1B). With regard to donor-derived chimerism in the recipient’s BM, there was no significant difference between *Bmal1*^*+/+*^ and *Bmal1*^*−/−*^-derived CD34^−^KSL cells (Figure 1E).

In a second competitive repopulation assay, at 12 weeks after the first BMT, 1 × 10^6^ BM cells from these recipients were transplanted into second recipient mice. At 4, 8 and 12 weeks after the second BMT, no big difference was also seen between the hematopoietic reconstitution ability of both donor-derived cells (Figure 1F and Additional file 1: Figure S1C). Moreover, we performed a third BMT at 12 weeks after the second BMT, but the result was the same as with the second BMT (data not shown).

### Normal frequencies and hibernation state of *Bmal1*^*−/−*^ HSCs

**Figure 2:**
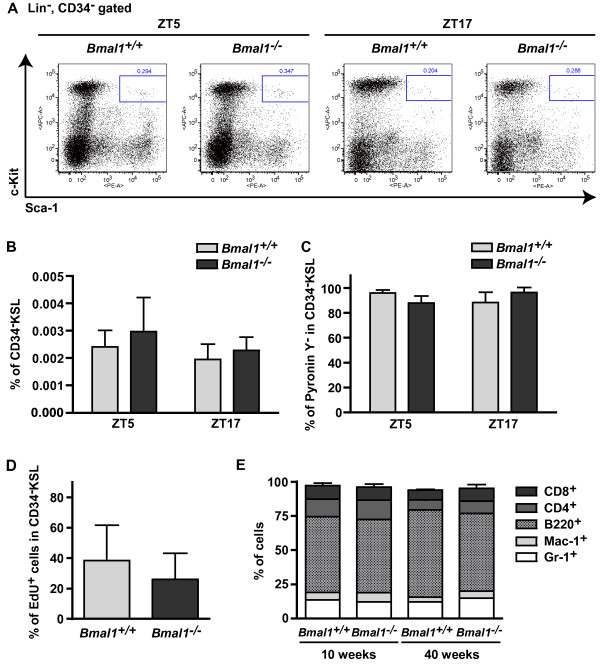
**Cell cycling and differentiation of HSCs are normal in arrhythmic *Bmal1 *deficient mice. A, B)** Normal frequency of HSCs in the BM of 8-10-week-old *Bmal1*^−/−^ mice. CD34^−^KSL fractions were assessed by flow cytometry. **A)** Data shown are representative of CD34^−^KSL cells at ZT5 and ZT17. **B)** The mean percentages ± SDs of CD34^−^KSL cells at ZT5 (n = 4) and ZT17 (n = 3) of two independent experiments. **C)** Comparable frequency of quiescent cells in HSC populations. HSCs of *Bmal1*^+/+^ and *Bmal1*^−/−^ mice were stained with Pyronin Y and analyzed by flow cytometry to give the mean percentages ± SDs of Pyronin Y^−^ cells in the CD34^−^KSL populations at ZT5 and ZT17 (n = 3) of two independent experiments. **D)** Normal EdU incorporation in *Bmal1*^−/−^ CD34^−^KSL cells. EdU was administered orally to mice for 3 weeks, and EdU incorporation into HSCs was evaluated using a Click-iT EdU PB Flow Cytometry Assay Kit. Data shown are the mean percentages ± SDs of EdU^+^ cells in HSC populations (*Bmal1*^−/−^ mice; n = 6, *Bmal1*^−/−^ mice; n = 3). **E)** White blood cell differentiation in young (10-week-old) and aged (40-week-old) mice. Each stack in the bar represents a cell type percentage. Gr-1^+^, granulocytes; Mac-1^+^, macrophages; B220^+^, B cells; CD4^+^, CD4^+^ T cells; and CD8^+^, CD8^+^ T cells (n = 6) of four independent experiments.

Although these results presented here led us to the conclusion that there appears to be no intrinsic circadian rhythm in HSCs, deficiency of *Bmal1* might change BM niche and affects the frequencies or cell cycling of HSCs. However, flow cytometry analysis of BM revealed no significant difference in the frequencies of *Bmal1*^*+/+*^ and *Bmal1*^*−/−*^ CD34^−^KSL cells at ZT5 and ZT17 (Figure 2A,B). Likewise, the frequencies of KSL cells, Common myeloid progenitor (CMP), Granulocyte-macrophage progenitor (GMP) and Megakaryocyte-erythroid progenitor (MEP) in *Bmal1*^*−/−*^ mice were similar to those in *Bmal1*^*+/+*^ mice (Additional file 2: Figure S2).

To investigate the hibernation status of HSCs in *Bmal1*^*−/−*^ mice, we stained CD34^−^KSL cells with Pyronin Y [12]. Consistent with our previous work [4], we found that most *Bmal1*^*+/+*^ and *Bmal1*^*−/−*^ CD34^−^KSL cells were negative for Pyronin Y staining, indicating normal HSC hibernation state, and that there were no differences depending on circadian rhythm (Figure 2C). In addition, after oral administration of EdU (5-ethynyl-2´-deoxyuridine) to *Bmal1*^*−/−*^ mice for 3 weeks, we could not obtain statistically significant difference in EdU incorporation between *Bmal1*^*+/+*^ and *Bmal1*^*−/−*^ CD34^−^KSL, indicating no alteration in cell cycling status (Figure 2D).

### *Bmal1* deficiency does not affect white blood cell differentiation

It has been reported that life span of *Bmal1*^*−/−*^ mice is only half that of wild-type mice [10], raising the possibility of an altered hematopoietic differentiation program in *Bmal1*^*−/−*^ mice. We therefore examined PB cells of *Bmal1*^*+/+*^ and *Bmal1*^*−/−*^ mice at 10 and 40 weeks of age. Although most *Bmal1*^*−/−*^ mice died within 40-week-old and the survived 40-week-old *Bmal1*^*−/−*^ mice looked older than their *Bmal1*^*+/+*^ counterparts, there were no significant changes in the levels of myeloid cells, B cells or T cells (Figure 2E).

## Concluding remarks

Recent studies have demonstrated that the central clock in suprachiasmatic nucleus (SCN) regulates the expression of *Cxcl12* through sympathetic nervous system [7] and *Cxcr4* expression in BM KSL cells or CD150^+^CD48^−^ cells [13] fluctuates according to circadian rhythms [14]. However, it has been reported that the clock genes are not expressed rhythmically in side population (SP) cells [15], suggesting that *Cxcr4* expression may be independent from control of clock genes. Moreover, Yagita et. al. [16] have recently found that circadian clock oscillation is not detected in mouse embryonic stem (ES) cells and induced pluripotent stem (iPS) cells, but is induced during their differentiation. Taken together, these findings appear to support the idea that the absence of circadian rhythm does not affect the function of stem cells in common.

In conclusion, despite the fact that mobilization of HSCs is controlled by circadian rhythm, our results demonstrate that *Bmal1* deficiency does not affect differentiation, proliferation and repopulating ability of murine HSCs. Therefore, we propose that circadian gene *Bmal1* is dispensable for intrinsic properties of murine HSCs.

## Abbreviations

HSC: Hematopoietic stem cell; BM: Bone marrow; PB: Peripheral blood; HSPCs: Hematopoietic stem and progenitor cells; BMT: Bone marrow transplantation; ZT: Zeitgeber time; CMP: Common myeloid progenitor; GMP: Granulocyte-macrophage progenitor; MEP: Megakaryocyte-erythroid progenitor; SCN: Suprachiasmatic nucleus; SP: Side population; ES: Embryonic stem; iPS: Induced pluripotent stem.

## Competing interests

The authors declare that they have no competing interests.

## Authors’ contributions

AI and SY designed the research and analyzed the data. AI, SY, SS and HN wrote the paper. All authors read and approved the final manuscript.

## Supplementary Material

Additional file 1: Figure S1 A) Traffic of HSPCs to bloodstream shows circadian oscillation. Circulating Colony-forming Units in Culture (CFU-Cs) did not oscillate in *Bmal1*^−/−^ mice (n = 3) compared with *Bmal1*^+/+^ mice (n = 4). Data shown are the mean percentages ± SDs of two independent experiments. B, C) Comparable long-term reconstitution ability of *Bmal1*^+/+^ and *Bmal1*^−/−^ HSCs during serial transplantation. Data shown are the mean ratios ± SDs of donor-derived cells in the PB at 4, 8 weeks after the first (n = 7) and the second BMT (n = 5) of three independent experiments. Click here for file

Additional file 2: Figure S2 Normal frequency of progenitors in the BM of 8-10-week-old *Bmal1*^−/−^ mice. KSL, CMP, GMP and MEP fractions were assessed by flow cytometry. The mean percentages ± SDs of KSL cells, CMP, GMP and MEP of *Bmal1*^+/+^ and *Bmal1*^−/−^ mice of two independent experiments (n = 3). Click here for file
