# Supplementary figures and images for: Clock gene Bmal1 is dispensable for intrinsic properties of murine hematopoietic stem cells
Source: J Negat Results Biomed. 2014 Mar 8;13:4. doi: 10.1186/1477-5751-13-4 (PMC4016489; doi:10.1186/1477-5751-13-4)

Supplemental Figure 1.

A

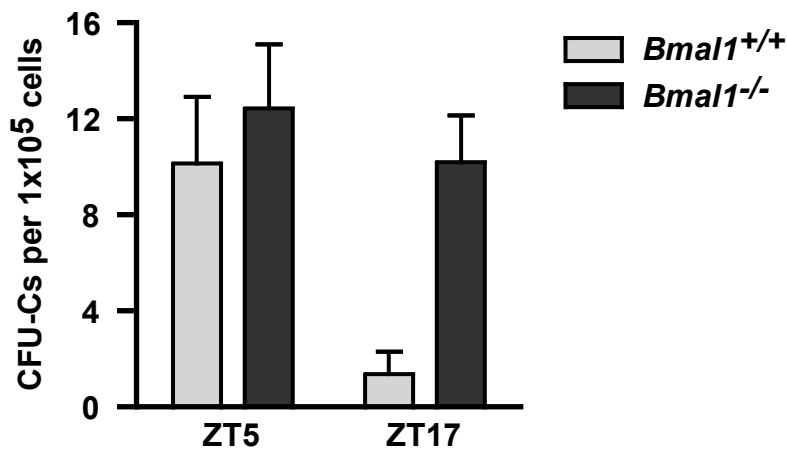

B

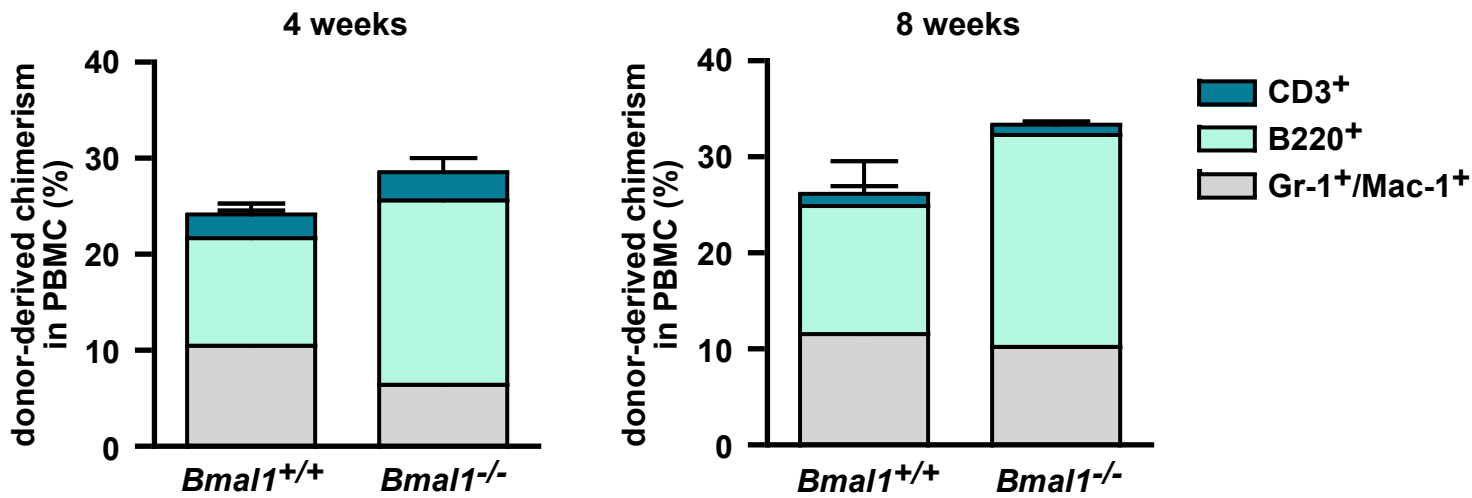

C

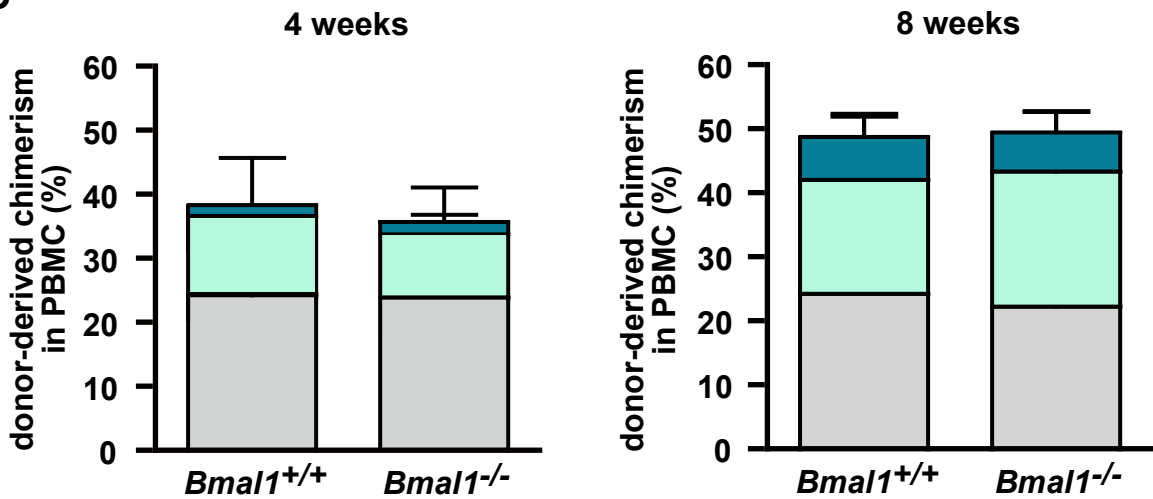

Supplement: Additional file 1: Figure S1 — A) Traffic of HSPCs to bloodstream shows circadian oscillation. Circulating Colony-forming Units in Culture (CFU-Cs) did not oscillate in Bmal1−/− mice (n = 3) compared with Bmal1+/+ mice (n = 4). Data shown are the mean percentages ± SDs of two independent experiments. B, C) Comparable long-term reconstitution ability of Bmal1+/+ and Bmal1−/− HSCs during serial transplantation. Data shown are the mean ratios ± SDs of donor-derived cells in the PB at 4, 8 weeks after the first (n = 7) and the second BMT (n = 5) of three independent experiments. [file 1477-5751-13-4-S1.pdf]

Supplemental Figure 2.

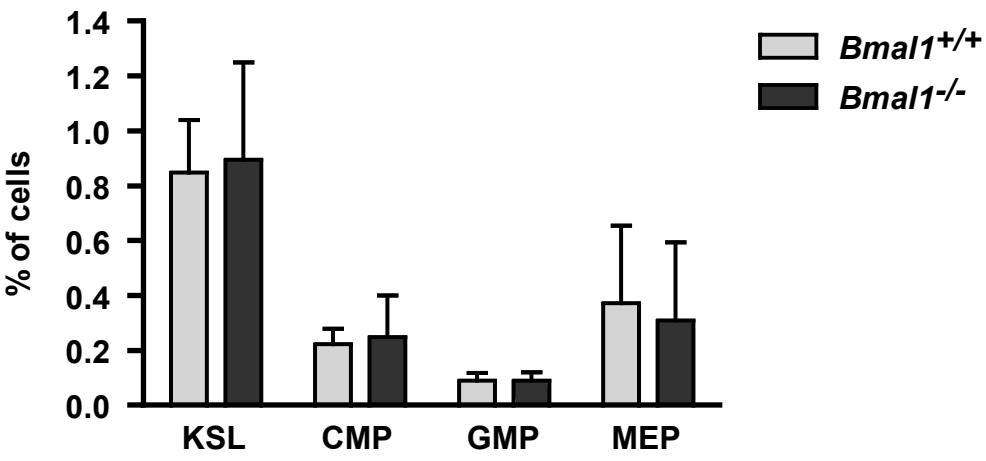

Supplement: Additional file 2: Figure S2 — Normal frequency of progenitors in the BM of 8-10-week-old Bmal1−/− mice. KSL, CMP, GMP and MEP fractions were assessed by flow cytometry. The mean percentages ± SDs of KSL cells, CMP, GMP and MEP of Bmal1+/+ and Bmal1−/− mice of two independent experiments (n = 3). [file 1477-5751-13-4-S2.pdf]
